# Investigation on Microstructures and Mechanical Properties of Isotactic Polypropylene Parts Fabricated by Different Process Conditions with Different Aging Periods

**DOI:** 10.3390/polym12122828

**Published:** 2020-11-28

**Authors:** Ying Liu, Tieli Zhu, Jie Bi, Weijian Hua, Tongmin Yu, Yifei Jin, Danyang Zhao

**Affiliations:** 1 Engineering Research Center for Molding Product of Ministry of Education, Dalian University of Technology, Dalian 116024, Liaoning, China; yingliu@dlut.edu.cn (Y.L.); zhutieli@dlut.edu.cn (T.Z.); jaybi1222@163.com (J.B.); yutmin@dlut.edu.cn (T.Y.); 2 School of Mechanical Engineering, Dalian University of Technology, Dalian 116024, Liaoning, China; 3 Mechanical Engineering Department, University of Nevada Reno, Reno, NV 89557, USA; weijianhua@nevada.unr.edu

**Keywords:** process parameters, ultrasound vibration, condensed microstructures, tensile strength, natural aging experiments

## Abstract

Polymeric parts have been increasingly used in various engineering fields. The performance of polymeric parts is significantly affected by working-environment-induced aging. In this paper, an ultrasonic-vibration-assisted injection molding system was designed and utilized to fabricate polymeric parts from isotactic polypropylene (iPP) using different processing conditions. The natural aging experiments were performed to age the fabricated iPP parts for one year. The effects of key process parameters as well as ultrasound power on the microstructures and the mechanical properties of the iPP parts after aging were systematically investigated using X-ray diffraction analysis, Fourier transform infrared analysis, scanning electron microscope imaging, and tensile testing. It is found that both the microstructures and the tensile strength of the iPP parts deteriorate with the increasing aging time. In addition, the crystallinity and the tensile strength decrease with the increasing melt temperature but increase with the increasing mold temperature in a given range and holding pressure. The increase in ultrasound power leads to an increase in crystallinity. However, when the ultrasound power is over 200 W, the tensile strength of the aged iPP parts decreases, which indicates that high ultrasound power may not form optimal condensed microstructures with excellent anti-aging capacity.

## 1. Introduction

Polymeric parts have been widely used in different engineering fields such as aerospace, automotive, naval architecture, electrical industry, and construction. The properties of polymeric parts are significantly affected by working-environment-induced aging, which may deteriorate the performance of the parts and even cause dysfunctionality [1,2,3]. Aging of polymeric parts is attributed to the inherent condensed microstructures, which significantly depend on the process conditions [4,5,6,7]. For polymers with the same formula and molecular structures, processing under different conditions, including process parameters and external fields, may result in completely different condensed microstructures and make the polymeric parts have different mechanical properties after aging [8]. As a result, understanding the effects of process parameters and external fields on these properties is of great significance, which can provide theoretical guidance to optimize the condensed microstructures and notably improve the mechanical properties of the fabricated polymeric parts after aging for a long time.

To date, numerous investigations have been performed to enhance the anti-aging performance of polymeric parts by either developing new material formulas or improving molecular structures. For example, Leong et al. [9] investigated the anti-aging performance of polypropylene and found that coupling between SiH_4_ and titanate can enhance the anti-aging performance of polypropylene filled with CaCO_3_ and talc. Li et al. [10] investigated the natural photoaging degradation of polypropylene (PP) and its nanocomposites. They found that PP nanocomposites with additives such as CaCO_3_ and SiO_2_ were more sensitive to photodegradation. Obadal et al. [11] studied the effects of ultraviolet (UV) irradiation on neat and β-nucleated isotactic polypropylene (iPP) and found that β-phase content in the crystallinity portion of β-PP remained stable under UV irradiation, which made β-nucleated iPP present lower degradability during photoaging. Výchopňová et al. [12] studied the effects of polymorphism on the photodegradation of iPP. In their study, they modified iPP by α-nucleating agent and β-nucleating agent and found that different crystalline morphologies can cause different photoaging resistances. Although the crystallinities of the samples were the same, the anti-photoaging ability of the samples with higher β-crystal content was better than that of the samples with higher α-crystal content. Nguyen et al. [13] investigated the UV weathering performance of high-density polyethylene-based composites with basalt fiber (BF)-reinforced shell. Their results revealed that the combination of BF and UV326 presented a synergistic effect on the alleviation of the photo-oxidation of wood–plastic composite shell layers. In summary, the aforementioned achievements on the properties of polymeric parts after aging were mainly obtained from materials. From the manufacturing perspective, there is a lack of studies that unveil the effects of process conditions. Since process conditions directly determine the microstructures of polymeric parts during fabrication, which further affect the mechanical properties after aging, it is necessary to investigate the effects of various process parameters.

Ultrasonic vibration has been introduced into injection molding since 1981 to fabricate parts from various polymers such as ultrahigh molecular weight polyethylene [14], polylactide [15], and isotactic polypropylene [16]. During ultrasonic vibration-assisted injection molding, the ultrasonic vibration field can affect the rheological properties of polymer melts, which further influence the crystallization of polymers in cooling, resulting in the formation of improved condensed microstructures. Thus, polymeric parts fabricated via ultrasonic vibration-assisted injection molding usually have better mechanical properties. However, the effects of the ultrasonic vibration field on the microstructures and mechanical properties after aging are still unknown.

Therefore, an ultrasonic vibration-assisted injection molding system was designed and utilized in this study to fabricate polymeric parts from isotactic polypropylene (iPP). The effects of the key process parameters (including melt temperature, mold temperature, and holding pressure) as well as the external ultrasonic vibration field on the microstructures and mechanical properties after aging have been systematically investigated. Herein, two factors, (1) microstructures and (2) mechanical properties, were selected to assess the properties of the fabricated iPP parts with different aging periods. The microstructures, including crystallinity, crystal forms, molecular structures, and surface morphologies were observed and analyzed using X-ray diffraction (XRD) analysis, Fourier transform infrared (FTIR) analysis, and scanning electron microscope (SEM) imaging, respectively, to explain the behaviors of the iPP parts after aging from the microscopic aspect. Tensile strength, one of the important mechanical properties, was measured by tensile testing to quantify the properties of iPP parts after aging from the macroscopic aspect. The aim of this study is to bridge the process parameters and the external field during injection molding with the microstructures and mechanical properties of the products after aging, which provides a feasible technical solution to achieve iPP parts with excellent anti-aging capacity.

## 2. Materials and Methods

### 2.1. Injection Molding Experiments

#### 2.1.1. Parts Design and Materials

**Figure 1:**
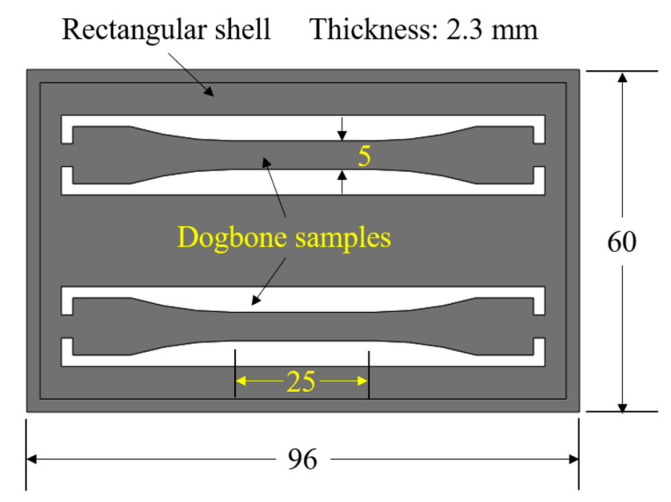
Structure and key dimensions of the designed part consisting of a rectangular shell and two dogbone-shaped samples. (Unit: mm.).

In this study, injection molding was used as the polymer processing approach to fabricate an iPP part, which was composed of a rectangular shell and two standard tensile test samples with dogbone morphology as shown in Figure 1. In particular, the dogbone samples had a gauge length of 25 mm, a gauge width of 5 mm, and a thickness of 2.3 mm. The polymer used for injection molding was isotactic polypropylene (T30S, West Pacific Petrochemical Company Ltd., Dalian, China) with an isotacticity of above 97%, a density of 1.15 g/cm^3^, and a melt flow index of 2.5 g/10 min.

#### 2.1.2. Injection Molding System and Experimental Design

**Figure 2:**
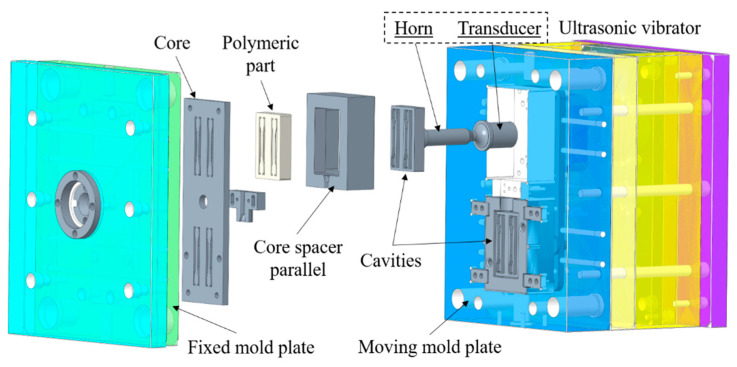
Schematic of ultrasonic vibration-assisted injection molding system. Ultrasonic vibration was applied to the injection mold during the injecting and holding stages.

An injection machine (model: SE100EV-C360, Sumitomo Heavy Industries, Ltd., Tokyo, Japan) was used as the core equipment of the injection molding system. A chiller (model: SPIA-05, Shenyang Jiaguang Machinery Equipment Co. Ltd., Shenyang, China) and a mold temperature control machine (model: MT-6L/6H, Shantou Xinyi Polymer Machinery Co. Ltd., Shantou, China) were used to cool down the mold after injection and control the mold temperature during injection, respectively. The injection mold was designed to have two cavities sharing one side gate. Two sets of ultrasonic vibration systems (tailormade by Shanghai Shengxi Supersonic Instrument Co., Ltd., Shanghai, China) were integrated with the mold to provide ultrasonic energy to the polymer melts in two cavities during the injection molding process as shown in Figure 2. The entire processing cycle was 35 s, including the injecting and holding stages (10 s with applied ultrasonic vibration) and the cooling stage (25 s without ultrasonic vibration).

**Figure 3:**
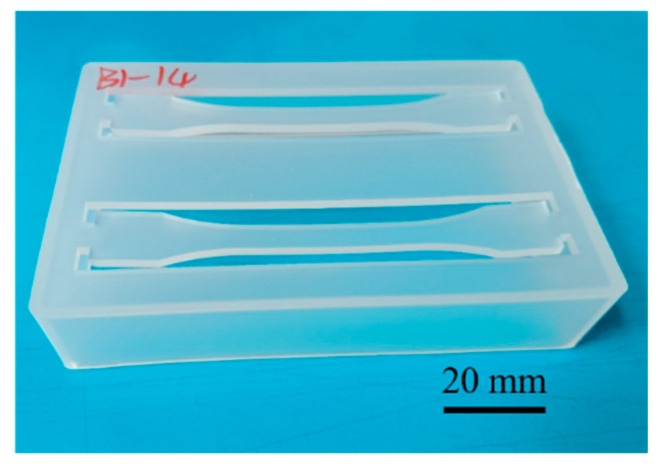
Representative isotactic polypropylene (iPP) part fabricated using the melt temperature (*T_1_*) of 220 °C, mold temperature (*T_2_*) of 50 °C, injection speed of 70 mm/s, injection pressure of 50 MPa, and holding pressure (*P*) of 44 MPa.

During injection molding, five process parameters can affect the formation of iPP parts with different microstructures and mechanical properties after aging. Based on the preliminary experimental results, the values of these five process parameters were determined as melt temperature (*T_1_*) of 220 °C, mold temperature (*T_2_*) of 50 °C, injection speed of 70 mm/s, injection pressure of 50 MPa, and holding pressure (*P*) of 44 MPa to successfully fabricate iPP parts with well-defined geometries as shown in Figure 3. Moreover, three process parameters (*T_1_, T_2_*, and *P*) were selected along with ultrasonic vibration (*P_w_*) with a frequency of 25 kHz to purposively design the experiments and systematically investigate their effects on the microstructures and mechanical properties of the iPP parts with different aging periods. The range of each parameter is illustrated in Table 1.

#### 2.1.3. Natural Aging Experiments

The fabricated iPP parts were naturally aged in a standard natural aging condition based on GB/T3681-2011. The parts were fixed on a homemade frame made from stainless steel and aged for 12 months. The samples before aging, after 6-month aging, and after 12-month aging were selected for testing.

### 2.2. Characterization of Fabricated iPP Parts

#### 2.2.1. X-ray Diffraction Tests

X-ray diffraction tests were performed using a X-ray diffractometer (model: D/Max-2400, Rigaku Co., Tokyo, Japan). During testing, the scanning speed was set as 8°/min, and the scanning range was from 5° to 50°. The data from XRD tests was analyzed by the software Jade 6.0 (Jade Software Co., Ltd., Christchurch, New Zealand) to obtain the XRD spectrums, which were further calculated in Jade 6.0 to achieve the crystallinities of the samples before and after aging.

#### 2.2.2. Fourier Transform Infrared Analysis

Fourier transform infrared analyses were conducted using an advanced Fourier transform infrared spectrometer (model: 6700, Thermo Fisher, Waltham, MA, USA) to explore the microscopic molecular structures of the samples before and after aging. During testing, the interactive K–K calibration method was used, and each sample was scanned 32 times with a resolution of 4. The maximum range of the optical table was set as 7.

#### 2.2.3. Scanning Electron Microscope Imaging

Scanning electron microscope imaging was performed to observe and image the surface morphology of the samples before and after natural aging. The samples were treated by gold spraying before testing and then imaged using a scanning electron microscope (model: Q45, FEI Co., Hillsboro, OR, USA) with a testing voltage of 20 kV and magnification of 2000.

#### 2.2.4. Tensile Tests

Tensile tests were performed using a tensile tester (model: 5965, Instron, Norwood, MA, USA) to the dogbone samples at room temperature for investigating their mechanical properties before and after aging. The tests were designed on the basis of GB/T 1040-2006 with a tensile rate of 100 mm/min. Each test took no more than 5 min. The load–position curve for each sample was obtained and used along with the geometries of the sample to calculate the stress–strain curve and achieve the tensile strength. It is noted that the difference between the aging temperature and the test temperature may affect the accuracy of the test results. To minimize the effects of temperature difference, each sample was moved from the aging environment to the lab and the tensile test was performed on this sample immediately.

#### 2.2.5. Statistical Analysis.

All quantitative values of the measurements in the XRD tests and tensile tests are reported as means ± standard deviation (SD) with *n* = 5 samples per group.

## 3. Results

### 3.1. Effects of Process Conditions on Crystallinity

**Figure 4:**
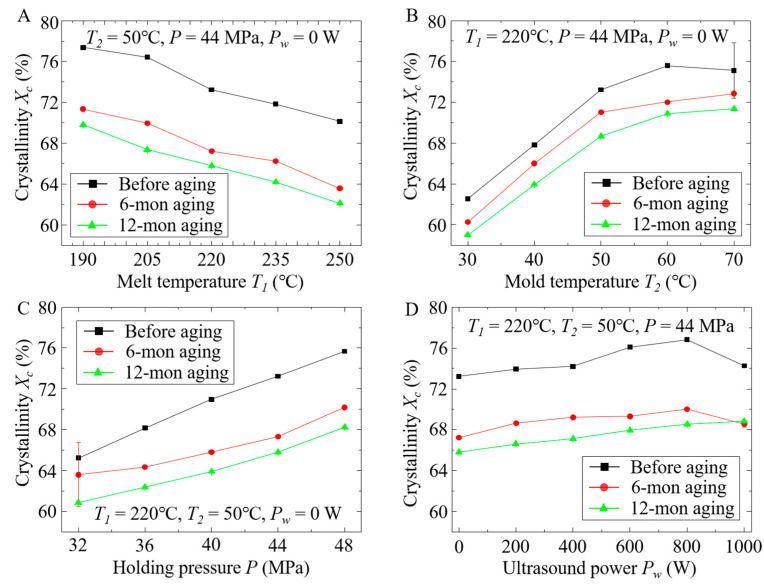
Effects of (**A**) melt temperature, (**B**) mold temperature, (**C**) holding pressure, and (**D**) ultrasound power on the crystallinity of the iPP samples before aging, after 6-month aging, and after 12-month aging.

The effects of process conditions on the crystallinity of the iPP parts were investigated, and the results are illustrated in Figure 4. From Figure 4A, it is found that the samples before aging had the highest crystallinity. The crystallinity of the samples after 6-month aging decreased by around 9%, and the crystallinity of the samples after 12-month aging decreased by around 12%. In addition, with the increase in melt temperature, crystallinity for each sample decreased. This phenomenon can be explained by the following reasons. First, when the melt temperature is relatively low, the viscosity of polymer melts is high. Thus, during mold filling, the shear stress gradient increases, which causes the orientation of inflowing polymer molecules to generate crystal nuclei that further grow into more crystals [17,18]. However, with the increase in melt temperature, the activity of polymer molecules increases, and the thermal motion continuously intensifies, which can make the distance between the molecular chains increase. As a result, it is difficult to form stable crystal nuclei or let the initial crystal nuclei proliferate into more crystals due to the violent thermal motion-induced chaos of molecular conformation [19]. Second, the increase in melt temperature leads to a decrease in viscosity, which weakens the shearing effect during mold filling, reduces the molecular orientation, and enhances the relaxation and curling capacity of polymer molecular chains, inhibiting the generation of crystal nuclei [17,18]. Finally, high melt temperature also enhances the relaxation and de-orientation of polymer molecules during the part-cooling process, which is harmful to the formation of regular and orderly arrangements of molecules [20]. Thus, melt temperature must be accurately controlled during injection molding to improve the crystallinity of the fabricated iPP parts.

The effects of mold temperature on the crystallinity of the samples are illustrated in Figure 4B. It is found that the crystallinity of the samples after 6-month aging decreased by around 3%, and that of the samples after 12-month aging decreased by around 7% compared with the crystallinity of the samples before natural aging. In addition, the crystallinity of the parts increased with the increasing mold temperature. This is because the increase in mold temperature can reduce the temperature difference between the polymer melts and the mold cavities, which can slow down the cooling speed of polymer melts in the cavities, maintaining a suitable temperature range for molecular crystallization for a relatively long time [21]. Furthermore, during the slow cooling process, molecules around the crystal nuclei can rapidly discharge into the crystal lattice, leading to the formation of more crystalline structures and the growth of more crystals [22]. As a result, the crystallinity in the iPP parts increases.

The effects of holding pressure on the crystallinity of the samples before and after aging are illustrated in Figure 4C. It is found that the crystallinity of the samples after 6-month aging decreased by around 8%, and that of the samples after 12-month aging decreased by around 11% compared with the crystallinity of the samples before natural aging. Moreover, with the increase in holding pressure, the crystallinity of the samples before and after aging increased gradually. This is because increasing the holding pressure can reduce the free volume of polymer molecules in the cavities, compress the arrangements between amorphous molecules and crystals, and densely squeeze the molecular chains [23]. Additionally, the increase in holding pressure also improves the orientation and arrangement of molecular chains, which makes it easier to generate more crystal nuclei and crystals [23,24], leading to an increase in the sample density. Therefore, increasing holding pressure can help linearly increase the crystallinity of the samples before and after aging, as shown in Figure 4C.

The effects of ultrasound power on the crystallinity of the samples before and after aging are illustrated in Figure 4D. It is found that, compared with the samples before aging, the crystallinity of the samples after aging decreased. In particular, the crystallinity of the samples after 6-month aging decreased by around 10%, and that of the samples after 12-month aging decreased by around 13%. In addition, with the increase in ultrasound power, the crystallinity of each sample gradually increased and then decreased. This is because the viscosity of polymer melts is relatively low under the action of the ultrasonic vibration field at a low power [25,26]. Thus, the shearing effect during mold-filling is strong, which benefits the orientation of molecular chains to generate crystals with regular shapes. However, the mechanical shocking effect caused by the high-frequency ultrasonic vibration makes polymer molecules difficult to quickly discharge into crystal lattices or even leads to crystal damage [27]. As a result, the crystallinity of each sample increases slowly due to the competition between these two opposite effects. When the ultrasound power is higher than a critical value, e.g., 800 W in this study, more energy generated by the ultrasonic vibration field accelerates the violent thermal motion of polymer molecules, inhibiting the formation of stable and regular crystal structures. Furthermore, the higher ultrasound power makes the mechanical shocking effect exceed the shearing effect, bringing damages to the formed crystal structures and leading to a decreased crystallinity of each sample.

### 3.2. Comparison of Crystal Forms before and after Aging

**Figure 5:**
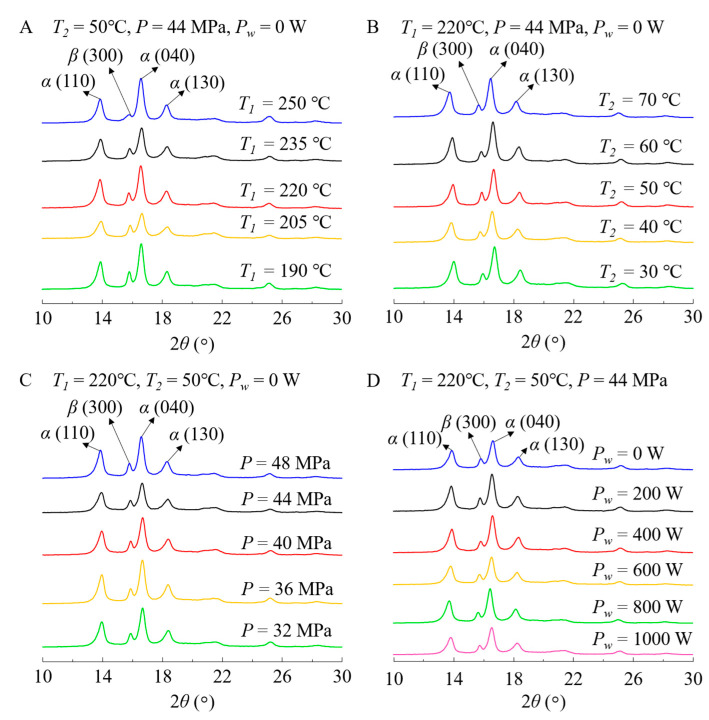
X-ray diffraction results of the iPP samples before aging. (**A**) Samples fabricated using different melt temperatures ranging from 190 to 250 °C, (**B**) samples fabricated using different mold temperatures ranging from 30 to 70 °C, (**C**) samples fabricated using different holding pressures ranging from 32 to 48 MPa, and (**D**) samples fabricated using different ultrasound powers ranging from 0 to 1000 W.

The crystal forms of the samples before natural aging can be seen in Figure 5. It is found that for all of the samples fabricated using different process conditions, the characteristic peaks of α-crystal can be observed at the locations of 13.96° (110), 16.70° (040), and 18.36° (130). Simultaneously, one characteristic peak of β-crystal was found at the location of 15.90° (300) [28,29]. As seen in Figure 5A, when the melt temperature was 190 °C, the characteristic peak of β-crystal had the highest value. In contrast, when the melt temperature increased to 250 °C, the characteristic peak of β-crystal decreased to the minimum. This phenomenon can be attributed to the lower crystallization temperature of β-crystal [30]. Thus, a lower melt temperature promotes the generation of β-crystals. From Figure 5B, it is found that at the mold temperature of 50 °C, the content of β-crystals in the samples was higher than those at other mold temperatures. In addition, it can be observed from Figure 5C that high holding pressure led to more β-crystals generated in the samples. This is because the increase in holding pressure causes the increase in shearing effect on the melt molecules in the cavities, which enlarges the crystallization temperature range of the β-crystal, resulting in the formation of more β-crystals during injection molding. Finally, the effects of ultrasound power on the crystal forms are illustrated in Figure 5D. It is found that when the ultrasound power was 200 W, the characteristic peak of β-crystal was slightly higher than those of the samples fabricated using other ultrasound powers. This can be explained by the molecular orientation during ultrasonic vibration. When the ultrasound power is relatively low, the molecular orientation can be improved in the ultrasonic vibration field, leading to the formation of molecular structures with the regular arrangement as well as more β-crystals [31]. However, with the increase in ultrasound power, the increasing ultrasonic vibration field enhances the thermal motion of molecules to be more chaotic [25], which inhibits the formation of β-crystals. In addition, the stronger ultrasonic vibration-induced mechanical impact brings damages to the molecular structures in the samples, which leads to the partial degradation of iPP [15]. Thus, the molecular orientation is weakened and is not conducive to the formation and growth of β-crystals. β-crystals directly affect the mechanical properties of the samples after aging. Usually, the samples with higher β-crystal content have higher tensile strength, higher impact strength, and better toughness. Moreover, the samples with higher β-crystal content have better transparency [32]. Thus, during natural aging, these samples transmit more and absorb less UV light, making them have better anti-photoaging capacity.

**Figure 6:**
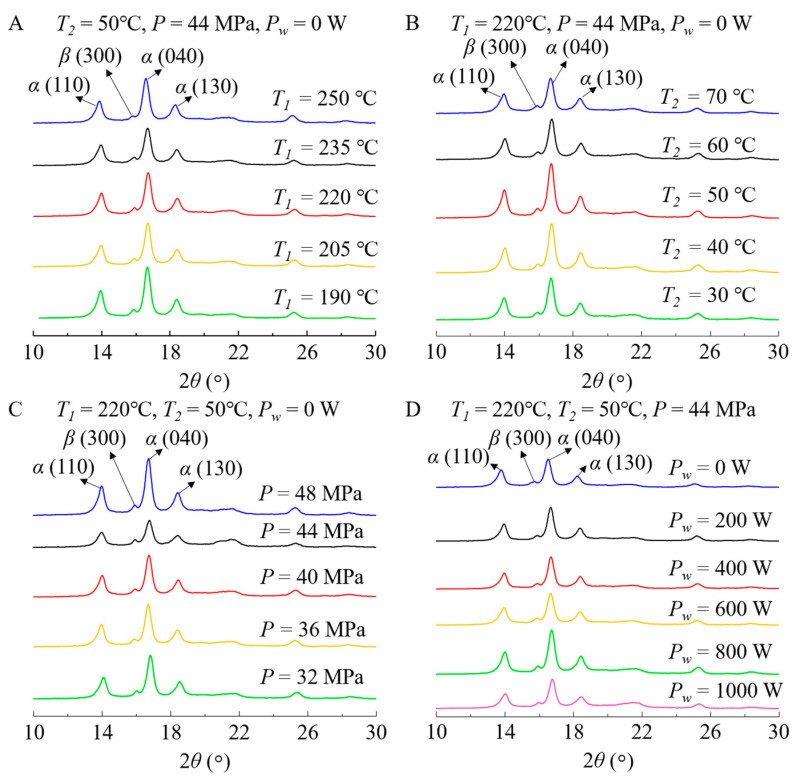
X-ray diffraction results of the iPP samples after 12-month aging. (**A**) Samples fabricated using different melt temperatures ranging from 190 to 250 °C, (**B**) samples fabricated using different mold temperatures ranging from 30 to 70 °C, (**C**) samples fabricated using different holding pressures ranging from 32 to 48 MPa, and (**D**) samples fabricated using different ultrasound powers ranging from 0 to 1000 W.

Figure 6 illustrates the crystal forms in the samples after 12-month aging. It is found that the characteristic peaks of both α- and β-crystals decreased compared with those in the samples before aging, indicating the decrease in the contents of α- and β-crystals after aging. Moreover, during natural aging, the UV spectrum in sunlight destroys C-C bonds in the iPP samples, so these bonds have reactions with oxygen and water molecules in air to form carbonyl (C=O) bonds and hydroxyl (-OH) groups [33], which damage the original molecular structures in the samples, destroys the crystal structures, and finally decreases the contents of both α- and β-crystals.

### 3.3. Comparison of Molecular Structures of the Samples before and after Aging

**Figure 7:**
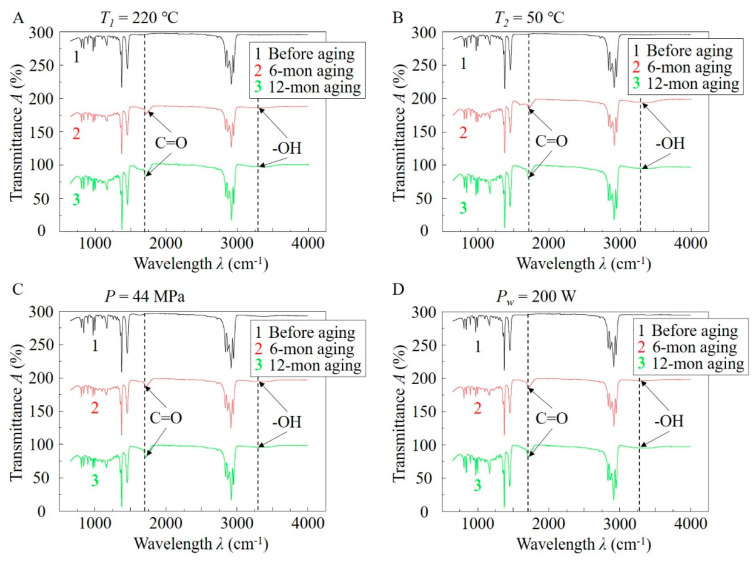
Fourier-transform infrared analysis of the iPP samples before aging, after 6-month aging, and after 12-month aging. The samples were fabricated using given (**A**) melt temperature of 220 °C, (**B**) mold temperature of 50 °C, (**C**) holding pressure of 44 MPa, and (**D**) ultrasound power of 200 W.

To explore the changes of the molecular structures in the samples before and after aging, FTIR analysis was performed on the samples fabricated using different process conditions as shown in Figure 7A–D, respectively.

As seen in Figure 7, the infrared spectrums of the samples presented pronounced changes before and after aging. In particular, the characteristic peak of the carbonyl group (C=O) appeared clearly in the interval of 1600–1900 cm^−1^ [34], which was the main absorption peak in most cases. Since there is no carbonyl group in iPP, the appearance of this characteristic peak indicates that the carbonyl group is generated in the molecular structures of the samples during natural aging. C-C bonds in iPP have a bond strength coincident with the energy of UV light (wavelength of 340–350 nm) in sunlight. As a result, the energy from UV light is completely absorbed by C-C bonds, leading to the breakup of the bonds. After that, carbon atoms oxidize with the oxygen in the environment and form C=O bonds, which is called UV-radiation-induced photo-oxidative aging [35]. The more pronounced characteristic peaks in the samples after 12-month aging demonstrated that more carbonyl groups were formed in these samples than those in the samples after 6-month aging. Thus, it is concluded that a longer natural aging period can lead to the formation of more broken C-C bonds and more carbonyl groups, severely damaging the original molecular structures in the samples.

Another change in the spectrums occurred at the location of approximately 3300 cm^−1^. It is found that compared with the samples before aging, a weaker characteristic peak of the hydroxyl group (-OH) appeared in the samples after aging, which generally presents in the spectrum interval of 3000–3500 cm^−1^ [33]. Since there is no hydroxyl group in iPP, this newly appearing characteristic peak is also due to the photo-oxidation during the natural aging process [33], in which C-C and C-H bonds in the original molecular structures break up and react with water molecules in the natural environment to form -OH groups. The more pronounced characteristic peaks in the samples after 12-month aging demonstrated that more hydroxyl groups were formed in these samples than those in the samples after 6-month aging. Although the characteristic peak of the hydroxyl group is weaker than that of the carbonyl group, the appearance of hydroxyl proves that in natural aging, not only does photo-oxygen reaction occur, but water molecules also play an important role in affecting the microstructures of the samples after aging.

From this analysis, it is concluded that natural aging can change the microstructures of the iPP parts from the molecular level. The breakup of original C-C and C-H bonds may react with oxygen and water molecules in the environment to form new microstructures or groups, leading to the change of condensed microstructures and mechanical properties after aging.

### 3.4. Comparison of Surface Morphologies of the Samples before and after Aging

**Figure 8:**
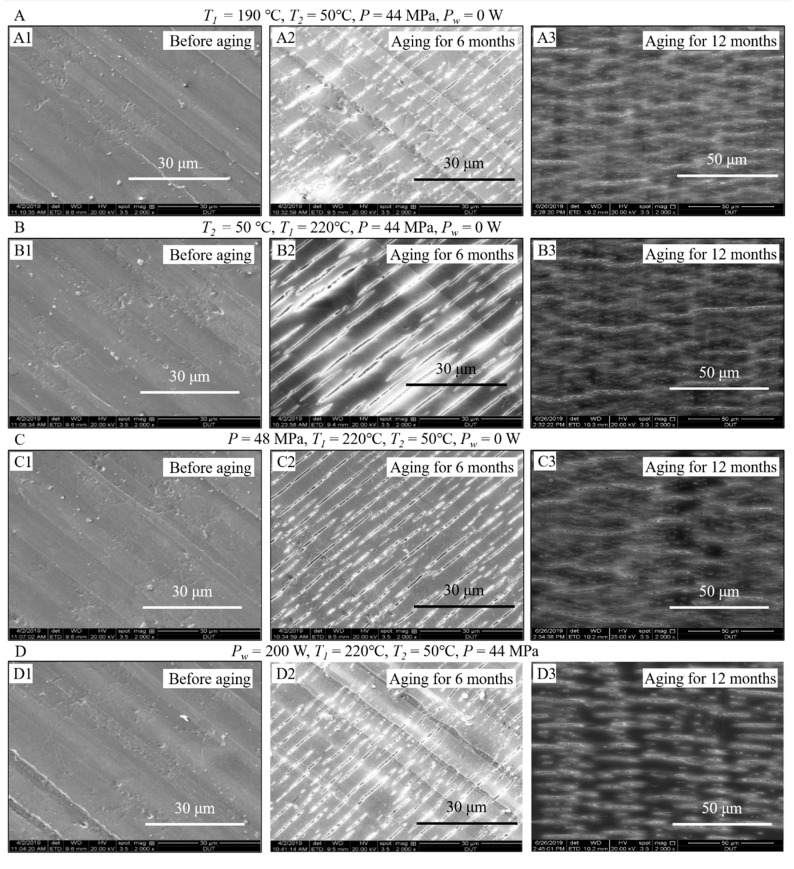
Scanning electron microscope images of the iPP samples before aging, after 6-month aging, and after 12-month aging. The samples were fabricated using (**A**) melt temperature of 190 °C, (**B**) mold temperature of 50 °C, (**C**) holding pressure of 48 MPa, and (**D**) ultrasound power of 200 W.

To explore the surface morphologies of the samples before and after aging, SEM imaging was performed on the samples fabricated using different process conditions as shown in Figure 8A–D, respectively. As shown in Figure 8, it is found that the surfaces of the samples before aging were relatively smooth. Except for several impurities, small dross, and pits, there were no obvious defects such as cracks (as shown in Figure 8A1–D1). In contrast, the surfaces of the samples after 6-month aging had poor quality with numerous cracks and pores, which oriented in mostly the same direction. For the samples after 12-month aging, the surface morphologies were even worse with condensed cracks and pores expanding in random directions, which indicates that natural aging severely affects the surface quality of the iPP parts.

The formation of cracks and pores in the samples can be explained as follows. UV radiation in sunlight induces the breakup of C–C and C–H bonds in the original molecular structures, which further form C=O groups by photo-oxygen reaction and –OH groups by reacting with water molecules in the environment. At the weak point where a molecular bond breaks, the intermolecular force decreases, leading to the generation of residual stress [36]. This stress orients along the injection direction and further causes the samples to expand and contract frequently in the changing natural environments, which finally results in the fatigue fracture (crack) on the surface of the samples in the direction perpendicular to the stress/injection direction [37]. After 6-month aging, because the stresses at the break of the internal molecular chains are aligned in the injection direction, the cracks formed are almost all aligned in the same direction, as shown in Figure 8A2–D2. Generally, the higher stresses lead to the formation of wider and deeper cracks, and vice versa. However, after 12-month aging, the number of broken molecular chains in the samples continues to increase and the degree of internal damages is severe, resulting in the chaotic arrangements of the molecules in the samples. Thus, the stress distributions are not limited to the orientation direction and the cracks start to extend in any directions where there are residual stresses generated by the breakup of molecular chains [37]. As a result, the damage degree of surface morphology continues to increase in the samples after 12-month aging as shown in Figure 8A3–D3.

### 3.5. Comparison of Tensile Behaviors of the Samples before and after Aging

**Figure 9:**
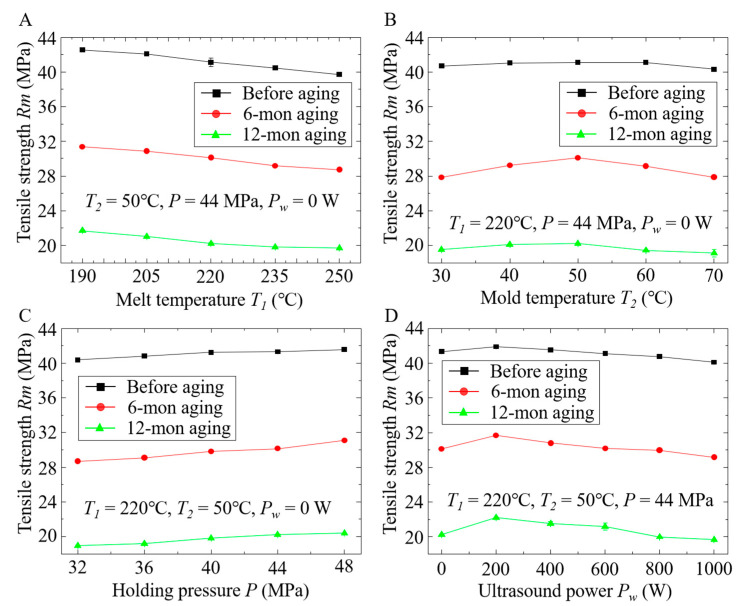
Effects of (**A**) melt temperature, (**B**) mold temperature, (**C**) holding pressure, and (**D**) ultrasound power on the tensile strength of the iPP samples before aging, after 6-month aging, and after 12-month aging.

The tensile tests were performed on the samples before and after natural aging, and the results are illustrated in Figure 9. Compared with the samples before aging, the samples after aging had relatively poor tensile strength. In particular, for the samples after 6-month aging, the tensile strengths of the samples fabricated by changing melt temperature, mold temperature, holding pressure, and ultrasound power decreased by 26%, 30%, 28%, and 27%, respectively. After 12-month aging, the tensile strengths decreased by 50%, 51%, 53%, and 48%, respectively, which indicates that natural aging has pronounced effects on the mechanical properties of the samples. It is noted that mechanical properties severely depend on the crystallinity of iPP parts [21]. With the increase in the aging period, the crystallinity of the samples fabricated under different conditions all decreased significantly, as shown in Figure 4. Thus, the decreasing crystallinity is the main factor causing the decreasing tensile strength. In addition, cracks were generated on the surface of the polymeric samples after aging for 6 months (Figure 8A2–D2) and 12 months (Figure 8A3–D3), which also resulted in the reduction of the tensile strength.

Figure 9A illustrates the relationship between melt temperature and tensile strength. It is found that the increase in melt temperature led to a decrease in the tensile strength of the samples before and after aging. In contrast, the increase in holding pressure resulted in an increase in the tensile strength as shown in Figure 9C, and the increasing rate of the samples after aging was slightly higher than that of the samples before aging. This can be attributed to the change of crystallinity under different conditions. As mentioned above, the increase in melt temperature resulted in a decrease in the crystallinity (Figure 4A), which further led to the decrease in tensile strength, while the increase in holding pressure benefited the formation of more crystals during injection molding, resulting in iPP parts with better mechanical properties.

In contrast with melt temperature and holding pressure, mold temperature and ultrasound power have different effects on the tensile strength of the samples. As shown in Figure 9B, with the increasing mold temperature, the tensile strength of the samples slightly increased, which was consistent with the previously reported results [38]. This is because, with the increase in mold temperature, the cooling speed of polymer melts during injection molding decreases, which provides a suitable environment for crystallization [21]. Thus, the crystallinity of the iPP parts increased, as shown in Figure 4B. However, with a higher mold temperature, crystallites can grow to larger size. This crystallinity versus crystallite size has combined effects on the mechanical properties of the fabricated parts, resulting in a slight change in tensile strength with the increasing mold temperature.

The effects of ultrasound power on the tensile strength are illustrated in Figure 9D. It is found that the tensile strength of the samples before and after aging first increased and then decreased. The tensile strength reached a maximum when the ultrasound power was ~200 W. This phenomenon can be explained by the content of β-crystals in the fabricated parts. It is noted that ultrasound has a selective effect on the growth of different crystal forms [31]. When the ultrasound power increases from 0 to 200 W, the crystallinity of β-iPP decreases, as reported in Ref. [31], although the crystallinity of entire iPP parts increases, as shown in Figure 4D. This decreasing crystallinity of β-iPP further leads to the increase in tensile strength, as reported in Ref. [39]. The decrease in the tensile strength of the iPP parts fabricated at higher ultrasound powers can be attributed to the cavitation phenomenon [40,41], in which the growth and collapse of microbubbles induce numerous shear forces to the polymer chains, causing polymer chain scission. With the increase in ultrasound power, both the number of microbubbles and their size increase, which leads to the increasing cavitation activity [14], damaging the microstructures of fabricated iPP parts. Thus, the effects of the cavitation on the mechanical properties exceed the influence of the crystallinity, resulting in the decrease in tensile strength in the high ultrasound power range (>200 W), as shown in Figure 9D.

## 4. Discussion

Natural aging has many factors that can affect the samples, such as light, oxygen, temperature, and moisture. In this paper, the natural aging period was selected as 12 months from May in the first year to April in the second year. This is because, in the first 6 months from May to October, the experimental location is in summer and fall, with relatively high temperature, and the locational solar irradiation is strong, with more UV rays in sunlight. In addition, summer and fall are two seasons with more precipitation and higher humidity. In contrast, the last 6-month aging occurred in the seasons of winter and spring, which have weaker solar irradiation. However, the atmospheric temperature in these two seasons is relatively low, especially in winter, with an atmospheric temperature below freezing point. Thus, the selected natural aging period can provide various representative aging conditions to validate the generalization of the obtained knowledge.

It is found that during the first 6-month natural aging period, UV radiation-induced photo-oxidative aging [35] is the leading factor due to the excellent solar irradiation, which damages the iPP molecular chains and destroys the crystal structures, resulting in a decrease in crystallinity (Figure 4), α- and β-crystal contents (Figure 6), and tensile strength (Figure 9). Higher humidity in this aging period also provides sufficient water molecules in the atmosphere. Thus, the broken C-C bonds by UV radiation on the main and branch chains can react with surrounding water molecules, further weakening the intermolecular force and decreasing the tensile strength. In the last 6-month natural aging period, the photo-oxidative aging effects deteriorate due to less solar irradiation in winter and spring. The low-atmosphere temperature makes the molecular chains in the samples frozen and difficult to move, resulting in brittle samples with poor mechanical toughness. Moreover, the atmospheric temperature changes back and forth around the freezing point in spring, making the molecular chains in the samples move actively at higher temperatures and freeze at lower temperatures. This continuous reciprocating cycle finally causes fatigue fracture of the molecular chains [37]. Although there is less UV radiation and precipitation in winter and spring, the inherent molecular structures are destroyed already during the first 6-month aging. The low-temperature environment and huge temperature difference further deteriorate the molecular structures in the samples. Therefore, due to the combined effects of the aforementioned factors, the microstructures and mechanical properties of the samples continue to decrease.

## 5. Conclusions

In this study, the effects of process conditions, including different process parameters and external ultrasonic vibration field, on the formation of condensed microstructures in the iPP parts and their tensile strengths before and after aging were systematically investigated. It is found that process conditions have great effects on the crystallinity and crystal forms of the iPP parts before and after aging. Natural aging can change the molecular structures in the iPP parts. With the increase in the aging period, the microscale surface morphologies of the iPP parts change from smooth surfaces with several impurities/dross/pits to surfaces with numerous oriented cracks and pores and finally to worse surfaces with randomly oriented condensed cracks and pores. Natural aging directly affects the mechanical properties of the iPP parts.

Since the mechanism of natural aging is extremely complicated, future work may focus on extending aging time and changing different aging conditions to investigate the effects of more natural factors on the microstructures and mechanical properties of polymeric parts, in particular, to understand the effects of photo-oxygen aging on the molecular structures in iPP parts. Future work will also include the design of orthogonal experiments to obtain the optimal combination of the operating parameters (including melt temperature, mold temperature, holding pressure, injection pressure, and injection speed), as well as the most suitable ultrasonic vibration field, in order to fabricate iPP parts with excellent microstructures and mechanical properties before and after aging. In addition, iPP was selected as an exemplary material in this study. In the future, the microstructures and mechanical properties of more commonly used polymers such as nylon, polyethylene, and poly (methyl methacrylate) after aging will be investigated.

## Figures and Tables

**Table 1:** Variables of single-factor experiments.

Process Parameters	Variables				
Melt temperature (°C)	190	205	220	235	250
Mold temperature (°C)	30	40	50	60	70
Holding pressure (MPa)	32	36	40	44	48
Ultrasound power (W)	200	400	600	800	1000

## References

[1] Hodge I.M. (1995). Physical aging in polymer glasses. Science.

[2] Celina M., Gillen K.T., Assink R.A. (2005). Accelerated aging and lifetime prediction: Review of non-Arrhenius behaviour due to two competing processes. Polym. Degrad. Stab..

[3] Celina M.C. (2013). Review of polymer oxidation and its relationship with materials performance and lifetime prediction. Polym. Degrad. Stab..

[4] Gulmine J.V., Akcelrud L. (2006). Correlations between structure and accelerated artificial ageing of XLPE. Eur. Polym. J..

[5] Roy P.K., Surekha P., Raman R., Rajagopal C. (2009). Investigating the role of metal oxidation state on the degradation behaviour of LDPE. Polym. Degrad. Stab..

[6] Ge S., Kang X., Zhao Y. (2011). One-year biodegradation study of UHMWPE as artificial joint materials: Variation of chemical structure and effect on friction and wear behavior. Wear.

[7] Matuana L.M., Jin S., Stark N.M. (2011). Ultraviolet weathering of HDPE/wood-flour composites coextruded with a clear HDPE cap layer. Polym. Degrad. Stab..

[8] Turnbull L., Liggat J.J., MacDonald W.A. (2012). Ageing of poly (ethylene terephthalate) and poly (ethylene naphthalate) under moderately accelerated conditions. J. Appl. Polym. Sci..

[9] Leong Y.W., Abu Bakar M.B., Mohd Ishak Z.A., Ariffin A. (2006). Filler treatment effects on the weathering of talc-, CaCO3-and kaolin-filled polypropylene hybrid composites. Compos. Interfaces.

[10] Li J., Yang R., Yu J., Liu Y. (2008). Natural photo-aging degradation of polypropylene nanocomposites. Polym. Degrad. Stab..

[11] Obadal M., Čermák R., Raab M., Verney V., Commereuc S., Fraïsse F. (2005). Structure evolution of α-and β-polypropylenes upon UV irradiation: A multiscale comparison. Polym. Degrad. Stab..

[12] Výchopňová J., Čermák R., Obadal M., Raab M., Verney V., Commereuc S. (2007). The role of specific nucleation in polypropylene photodegradation. Polym. Degrad. Stab..

[13] Nguyen V.D., Hao J., Wang W. (2018). Ultraviolet weathering performance of high-density polyethylene/wood-flour composites with a basalt-fiber-included shell. Polymers.

[14] Sánchez-Sánchez X., Hernández-Avila M., Elizalde L.E., Martínez O., Ferrer I., Elías-Zuñiga A. (2017). Micro injection molding processing of UHMWPE using ultrasonic vibration energy. Mater. Des..

[15] Sacristan M., Planta X., Morell M., Puiggali J. (2014). Effects of ultrasonic vibration on the micro-molding processing of polylactide. Ultrason. Sonochem..

[16] Li D., Xin Y., Song Y., Dong T., Ben H., Yu R., Zhang Y. (2019). Crystalline modification of isotactic polypropylene with a rare earth nucleating agent based on ultrasonic vibration. Polymers.

[17] Somani R.H., Yang L., Sics I., Hsiao B.S., Pogodina N.V., Winter H.H., Agarwal P., Fruitwala H., Tsou A. (2002). Orientation-induced crystallization in isotactic polypropylene melt by shear deformation. Macromol. Symp..

[18] Somani R.H., Yang L., Hsiao B.S., Fruitwala H. (2003). Nature of shear-induced primary nuclei in iPP melt. J. Macromol. Sci. Phys..

[19] Liu P., Hu A., Wang S., Shi M., Ye G., Xu J. (2011). Evaluation of nonisothermal crystallization kinetic models for linear poly (phenylene sulfide). J. Appl. Polym. Sci..

[20] Lee W.I., Talbott M.F., Springer G.S., Berglund L.A. (1987). Effects of cooling rate on the crystallinity and mechanical properties of thermoplastic composites. J. Reinf. Plast. Compos..

[21] Rizvi S.J.A. (2017). Effect of injection molding parameters on crystallinity and mechanical properties of isotactic polypropylene. Int. J. Plast. Technol..

[22] Ameli A., Nofar M., Jahani D., Rizvi G., Park C.B. (2015). Development of high void fraction polylactide composite foams using injection molding: Crystallization and foaming behaviors. Chem. Eng. J..

[23] Pantani R., Coccorullo I., Speranza V., Titomanlio G. (2007). Morphology evolution during injection molding: Effect of packing pressure. Polymer.

[24] Le M.C., Belhabib S., Nicolazo C., Vachot P., Mousseau P., Sarda A., Deterre R. (2011). Pressure influence on crystallization kinetics during injection molding. J. Mater. Process. Technol..

[25] Chen J., Chen Y., Li H., Lai S.Y., Jow J. (2010). Physical and chemical effects of ultrasound vibration on polymer melt in extrusion. Ultrason. Sonochem..

[26] Ávila-Orta C., Espinoza-González C., Martínez-Colunga G., Bueno-Baqués D., Maffezzoli A., Lionetto F. (2013). An overview of progress and current challenges in ultrasonic treatment of polymer melts. Adv. Polym. Technol..

[27] Zabihi F., Eslamian M. (2018). Effect of the ultrasonic substrate vibration on nucleation and crystallization of PbI2 crystals and thin films. Crystals.

[28] Yuan Y., Chen B., Zhang X. (2007). Study on the formation of β-crystal during the crystallization process of polypropylene reactor granule. Polymer.

[29] Chen C., Zhang Z., Ding Q., Wang C., Mai K. (2015). Influence of different β-nucleating agent on crystallization behavior, morphology, and melting characteristic of multiwalled carbon nanotube-filled isotactic polypropylene nanocomposites. Polym. Compos..

[30] Dou Q. (2008). Effect of the composition ratio of pimelic acid/calcium stearate bicomponent nucleator and crystallization temperature on the production of β crystal form in isotactic polypropylene. J. Appl. Polym. Sci..

[31] Kang J., Chen J., Cao Y., Li H. (2010). Effects of ultrasound on the conformation and crystallization behavior of isotactic polypropylene and β-isotactic polypropylene. Polymer.

[32] Zhang Z., Chen C., Wang C., Guo J., Mai K. (2011). Nonisothermal crystallization kinetics of isotactic polypropylene nucleated with a novel supported β-nucleating agent. J. Therm. Anal. Calorim..

[33] Madeleine-Perdrillat C., Delor-Jestin F., Bussiere P.O., de Sainte Claire P., Pilichowski J.F., Baba M. (2014). Simultaneous UV or thermal exposure and IR detection of evolved vapours: New tool for studying polymer photo-degradation. J. Photochem. Photobiol. A.

[34] He P., Xiao Y., Zhang P., Xing C., Zhu N., Zhu X., Yan D. (2005). Thermal degradation of syndiotactic polypropylene and the influence of stereoregularity on the thermal degradation behaviour by in situ FTIR spectroscopy. Polym. Degrad. Stab..

[35] Islam N.Z.M., Othman N., Ahmad Z., Ismail Z. (2011). Effect of pro-degradant additive on photo-oxidative aging of polypropylene film. Sains. Malays.

[36] Gupta S., McDonald B., Carrizosa S.B., Price C. (2016). Microstructure, residual stress, and intermolecular force distribution maps of graphene/polymer hybrid composites: Nanoscale morphology-promoted synergistic effects. Compos. B Eng..

[37] Lv Y., Huang Y., Yang J., Kong M., Yang H., Zhao J., Li G. (2015). Outdoor and accelerated laboratory weathering of polypropylene: A comparison and correlation study. Polym. Degrad. Stab..

[38] Vadori R., Mohanty A.K., Misra M. (2013). The effect of mold temperature on theperformance of injection molded poly (lactic acid)-based bioplastic. Macromol. Mater. Eng..

[39] Tordjeman P., Robert C., Marin G., Gerard P. (2001). The effect of α, β crystalline structure on the mechanical properties of polypropylene. Eur. Phys. J. E. Soft Matter.

[40] Peshkovskii S.L., Friedman M.L., Tukachinskii A.I., Vinogradov G.V., Enikolopian N.S. (1983). Acoustic cavitation and its effect on flow in polymers and filled systems. Polym. Compos..

[41] Pawlak A., Galeski A., Rozanski A. (2014). Cavitation during deformation of semicrystalline polymers. Prog. Polym. Sci..

